# The efficacy of re-challenge with BRAF inhibitors after previous progression to BRAF inhibitors in melanoma: A retrospective multicenter study

**DOI:** 10.18632/oncotarget.26149

**Published:** 2018-09-28

**Authors:** Julia K. Tietze, Andrea Forschner, Carmen Loquai, Heidrun Mitzel-Rink, Lisa Zimmer, Frank Meiss, David Rafei-Shamsabadi, Jochen Utikal, Maike Bergmann, Friedegund Meier, Nicole Kreuzberg, Max Schlaak, Carsten Weishaupt, Claudia Pföhler, Mirjana Ziemer, Michael Fluck, Jessica Rainer, Markus V. Heppt, Carola Berking

**Affiliations:** ^1^ Department of Dermatology and Allergy, University Hospital Munich (LMU), 80337 Munich, Germany; ^2^ Department of Dermatology, Center for Dermatooncology, University Hospital Tübingen, 72076 Tübingen, Germany; ^3^ Department of Dermatology, University Medical Center Mainz, 55131 Mainz, Germany; ^4^ Department of Dermatology, University Hospital Essen, University of Essen, 45147 Essen, Germany; ^5^ Department of Dermatology and Venereology, Medical Center - University of Freiburg, Faculty of Medicine, University of Freiburg, 79104 Freiburg, Germany; ^6^ Skin Cancer Unit, German Cancer Research Center (DKFZ) and Department of Dermatology, Venereology and Allergology, University Medical Center Mannheim, Ruprecht-Karl University of Heidelberg, 68167 Mannheim, Germany; ^7^ Department of Dermatology, Skin Cancer Center, National Center for Tumor Diseases, Medical Faculty and University Hospital Carl Gustav Carus, TU Dresden, 01307 Dresden, Germany; ^8^ Department of Dermatology, University of Cologne, 50937 Cologne, Germany; ^9^ Department of Dermatology, University of Münster, 48149 Münster, Germany; ^10^ Department of Dermatology, Saarland University Medical Center, 66421 Homburg/Saar, Germany; ^11^ Department of Dermatology, Venereology, and Allergology, University Hospital Leipzig, 04103 Leipzig, Germany; ^12^ Department of Internal Medical Oncology, Clinic Hornheide, 48157 Münster, Germany; ^13^ Deparment of Dermatology, Klinikum Süd, 86179 Augsburg, Germany

**Keywords:** melanoma, BRAF-inhibition, MEK-inhibition, re-challenge, BRAFV600 mutation

## Abstract

BRAF and MEK inhibition is efficient in patients with BRAF V600-mutated metastatic melanoma, but due to acquired resistance the duration of response (DoR) is often only short-lived. In this retrospective multicenter study with 60 patients suffering from inoperable or metastatic melanoma we evaluated the efficacy of re-challenge with a BRAF inhibitor (BRAF2) with or without MEK-inhibition after progressive disease upon previous treatment with a BRAF inhibitor (BRAF1) with or without MEK inhibition.

Treatment with BRAF1 led to a disease control rate (DCR) of 90% with 12% complete responses (CR), 58% partial responses (PR) and 20% stable diseases (SD), the median progression-free survival (PFS) was 9.9 and DoR 10.7 months. BRAF2 with (68%) or without (32%) additional MEK inhibition was initiated after a median interval of 3.4 months. DCR after re-challenge with BRAF2 was 57%, 8% CR, 20% PR and 28% SD, median PFS was 5.0 and DoR 14.0 months. The duration of the treatment interval or the treatment in the interval did not influence the DCR or PFS to BRAF2. The only predictive factor for response to BRAF2 was previous response to BRAF1; all patients with CR to BRAF1 achieved disease control with BRAF2, but only 60% of the patients with PR to BRAF1 (p=0.002). Addition of MEK inhibition to BRAF2 after treatment with BRAF1 as monotherapy did not significantly increase the DCR or PFS compared to patients treated solely with mono- or combination therapy.

In conclusion re-challenge with a BRAF inhibitor is a meaningful therapeutic option for patients with BRAF V600-mutated metastatic melanoma.

## INTRODUCTION

About 50% of patients with metastatic melanoma carry a mutation in the serine-threonine protein kinase B-RAF, mostly BRAF V600E or V600K. They are eligible for treatment with BRAF inhibition used generally in combination with mitogen-activated protein kinase (MEK) inhibition [1, 2]. The treatment is very effective with a disease control rate (DCR) of 90%; however, the median progression-free survival (PFS) is only about 7 months with BRAF inhibitor monotherapy and about 12 months with the combination of BRAF and MEK inhibitor therapy, but long-lasting responses have also been observed [1–4]. The limited PFS is caused by the development of resistance characterized by re-activation of the mitogen-activated protein kinase kinase (MAPK) signaling in 70% of the cases [5]. The molecular mechanisms include enhanced CRAF signaling [6], mutations in N-ras proto-oncogene (NRAS) [7], BRAF gene amplification [7], overexpression of the serine threonine kinase MAP3K8 [8], and elevation of platelet-derived growth factor receptor-β [9]. More recently it has been shown that Mer-tyrosine-kinase I is specifically upregulated in resistant melanoma cells [10].

It has been postulated that the composition of the tumor cells is a dynamic process, also depending -among other factors- on drug-induced changes in the microenvironment. Indeed, the coexistence of different resistance mechanisms was found in a patient who was successfully re-challenged with a BRAF inhibitor after treatment with chemo/immunotherapy [11]. In 2013 Thakur et al. published a mouse model with vemurafenib-resistant melanoma. They observed that resistant tumors showed continued dependency on BRAF (V600E) signaling due to elevated BRAF (V600E) expression [12]. These melanoma cells depended on the supply of a BRAF inhibitor and showed a fitness deficit in the absence of vemurafenib leading to regression. Sun et al. showed in 2014 that epidermal growth factor receptor expression on melanoma cells may be rapidly enhanced in the presence of a BRAF inhibitor. However, this is reversed when the drug is discontinued [9], which also led to a regression of the resistant cells. A drug holiday may therefore promote re-growth of cells, which are sensitive to BRAF inhibition, and hence may overcome resistance.

Based on these data and considering the limited treatment options after progression to both BRAF inhibition and immune checkpoint blockade (ICB), it seemed worthwhile to explore re-initiation of BRAF inhibition (re-challenge) as a relatively well-tolerated and convenient oncologic therapy.

So far only few groups have published data on re-challenge after progression under BRAF-inhibition in the clinical setting [13–17]. A recently published retrospective analysis and a prospective study of 25 melanoma patients support the experimental data and showed response after re-challenge with a BRAF inhibitor. Roux et al. published a case series with 10 patients in 2015 [14], Schreuer et al., published a prospective study on re-challenge after progress under dabrafenib and trametinib with 25 patient [15] and Valpione et al. published a retrospective analysis with 116 patients with re-challenge of BRAF inhibition [17]. However, in this study only 71.6% of the patients received the re-challenge with BRAF2 after progression under BRAF1, the other patients discontinued treatment with BRAF1 due to other reasons such as side effects. In the analysis these patients were not separately evaluated.

Here we present a retrospective analysis of 60 patients with inoperable or metastatic melanoma. These patients were re-challenged with a BRAF inhibitor (BRAF2) with or without additional MEK-inhibition after previous progression to BRAF inhibitor (BRAF1) treatment with or without additional MEK-inhibition. For the re-challenge the patient received either the previous used BRAF-inhibitor or a different BRAF-inhibitor.

## RESULTS

### Patient cohort

In total, 60 patients with advanced BRAFV600 mutated melanoma were included in the analysis. Ninety percent of the patients (54/60) were suffering from stage IV and 10% (6/60) from stage IIIc melanoma. The median age was 56 (28-79) years and the majority, 60% (36/60), was male. Of cutaneous origin were 83% (50/60) of the melanoma, 3% (2/60) originated from mucosa and in 13% (8/60) of the cases the primary location was unknown.

Before initiation of the treatment with BRAF1, in median three organ systems were affected by metastases.

The Eastern Cooperative Oncology Group (ECOG) performance before treatment start with BRAF1 was 0 in 78% (47/60), 1 in 18% (11/60) and 2 in 2% (1/60) of the patients, in 2% (1/60) the performance was unknown.

Before treatment start increased serum levels of serum protein S100 were detected in 70% (42/60) and increased serum levels of lactate dehydrogenase (LDH) in 57% (34/60) of the patients. Brain metastases were diagnosed in 18% (11/60) of the patients (Table 1), 64% of these (7/11) received radiation, 2 whole-brain irradiation and 5 stereotactic radiosurgery, and one surgery before initiation of BRAF1. Pretreatment with ipilimumab was conducted in 10% (6/60) of the patients.

**Table 1:** Demographic, response and treatment data of patients treated with BRAF inhibitor 1 (BRAF1) and BRAF inhibitor 2 (BRAF2) as re-challenge

		BRAF1		BRAF2	
		No	%	No	%
**Dosage of BRAF inhibitor**	100%	57	95%	57	95%
	75%	2	3%	2	3%
	50%	1	2%	1	2%
**Combination with MEK inhibitor**		19	32%	41	68%
**BRAF inhibitor**	Vemurafenib	32	53%	13	22%
	Dabrafenib	16	27%	43	72%
	Encorafenib	12	20%	4	7%
**Number of involved organs**	1	6	10%	5	8%
	2	20	33%	14	23%
	3	16	27%	15	25%
	>3	18	30%	26	43%
	Liver met	19	32%	20	33%
	brain met	11	18%	36	60%
**Response**	CR	7	12%	5	8%
	PR	35	58%	12	20%
	SD	12	20%	17	28%
	MR	2	3%	7	12%
	PD	4	7%	19	32%

### Treatment with BRAF1

**Figure 1:**
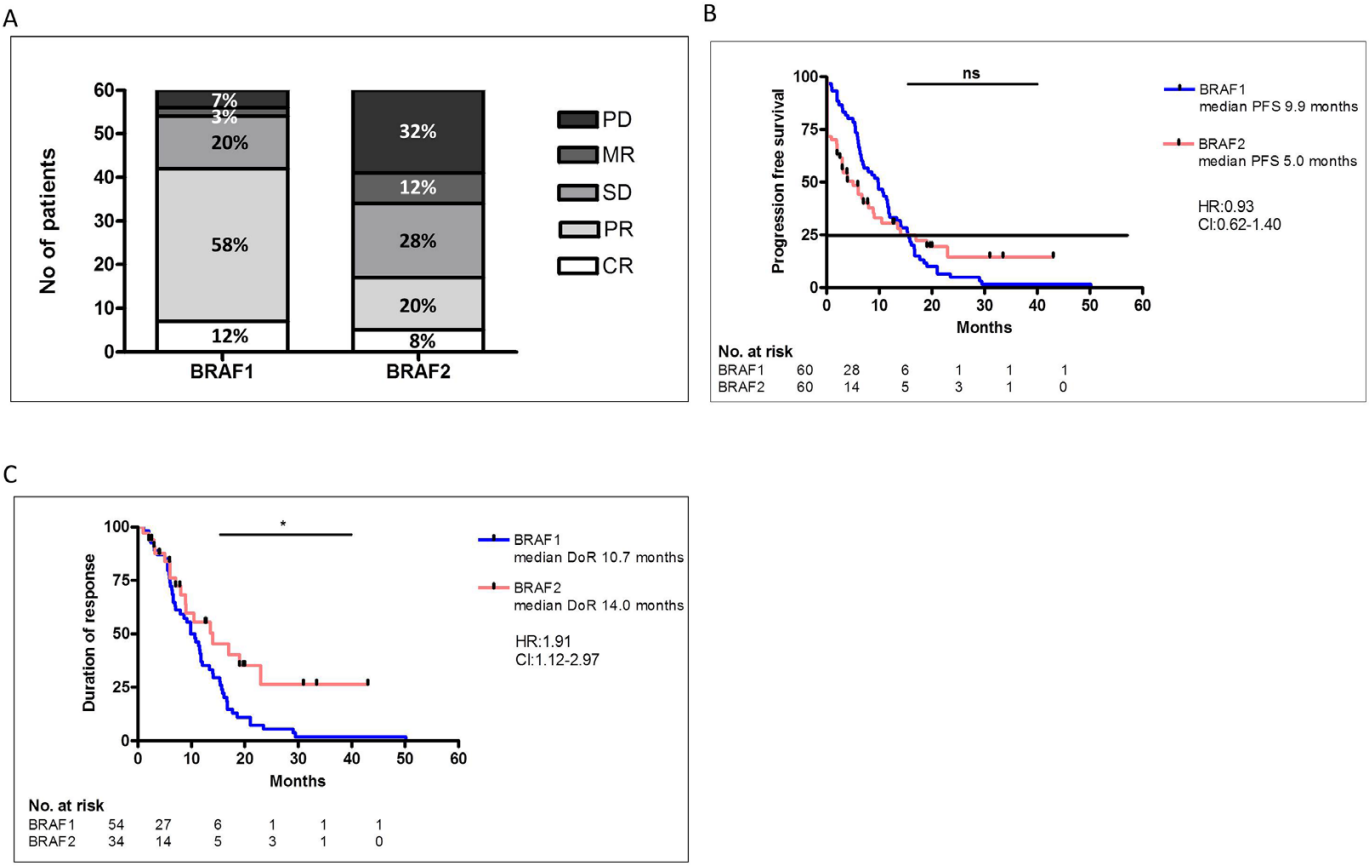
Comparison of response rates of initial BRAF inhibitor (BRAF1) therapy and of re-challenge with BRAF inhibitor 2 (BRAF2) **(A)**. There was no significant difference in the PFS with BRAF1 or BRAF2 **(B)**. The DoR was significantly higher after response to BRAF2 compared to BRAF1 **(C)**.

Most patients (80%) received BRAF inhibition as a first-line treatment. Treatment with BRAF inhibitors was initiated between November 2010 and December 2015. In 92% (55/60) of the patients a BRAF V600E mutation was detected. The majority of the patients received BRAF1 inhibition as monotherapy, only 32% (19/60) of the patients were treated with a combination of BRAF- and MEK-inhibition. The disease control rate (DCR) after BRAF1 was 90%, with 12% (7/60) CR, 58% (35/60) PR and 20% (12/60) SD (Figure 1A). In 10% of the patients the disease was not controlled by treatment with BRAF1 regardless of the combination with a MEK-inhibition. 3% (2/60) of these patients showed mixed responses (MR) and 7% (4/60) progressive disease (PD) (Figure 1A). The median PFS was 9.9 months (Figure 1B) and the duration of response (DoR) was 10.7 months (Figure 1C).

### Treatment in the interval between BRAF1 and BRAF2

The median duration of the interval after BRAF1 was terminated and before BRAF2 was initiated was 3.4 (0-17.8) months. In the interval 67% (40/60) of the patients received ICB. Of these 27% (16/60) were treated with PD-1 blockade, 22% (13/60) with ipilimumab, 7% (4/60) with a combination of ipilimumab and nivolumab. Eight percent (5/60) of the patients received chemotherapy and ICB. Local therapy only, i.e., radiation or surgical therapy was applied in 8% (5/60) of the patients. Thirteen patients, 22% (13/60), were switched from BRAF1 to BRAF2 without a different treatment in between, most of them within clinical trials.

**Figure 2:**
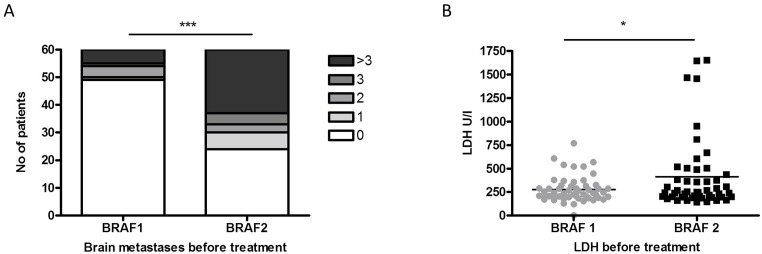
The number of patients diagnosed with brain metastases **(A)** and with increased LDH levels **(B)** differed significantly before re-challenge with BRAF inhibitor (BRAF2) compared to initial therapy with BRAF inhibitor 1 (BRAF1).

When comparing the health status of the patients before initiation of BRAF1 and before initiation of BRAF2 signs of disease progression could be observed. Thus, the number of patients affected with brain metastases (36/60) (p<0.0001) and the median serum level of LDH (p=0.019) had increased significantly (Figure 2A, 2B). Radiation therapy, specifically 6 whole-brain irradiations and 4 stereotactic radiosurgeries, was performed in 38% (10/26) of the patients with newly diagnosed brain metastases before treatment start or during treatment with BRAF2.

The Eastern Cooperative Oncology Group (ECOG) performance before treatment start with BRAF2 was significantly worse compared to BRAF1 with 0 in 28% (17/60), 1 in 35% (21/60), 2 in 22% (14/60), 3 in 8% (5/60) of the patients (p<0,0001), in 5% (3/60) the performance was unknown.

The number of affected peripheral organ systems and the level of S100 did not differ significantly before treatment start of BRAF2 compared to treatment start of BRAF1.

### Re-challenge with BRAF2

Despite detectable disease progression, most patients benefited from the re-challenge with BRAF2. DCR after re-challenge with BRAF2 with or without MEK-Inhibition was 57% (34/60), with 8% (5/60) complete responses (CR), 20% (12/60) partial responses (PR) and 28% (17/60) stable diseases (SD) (Figure 1A). In 68% (41/60) of the patients BRAF2 was combined with a MEK-inhibition. The median PFS under BRAF2 with our without MEK-inhibition was 5.0 months. PFS under BRAF1 and PFS under BRAF2 did not differ significantly (Figure 1B). DoR under BRAF2 was 14.0 months, which was significantly longer than DoR under BRAF1 (p=0,016) (Figure 1C).

### Analysis of potentially prognostic factors for response to BRAF2

**Figure 3:**
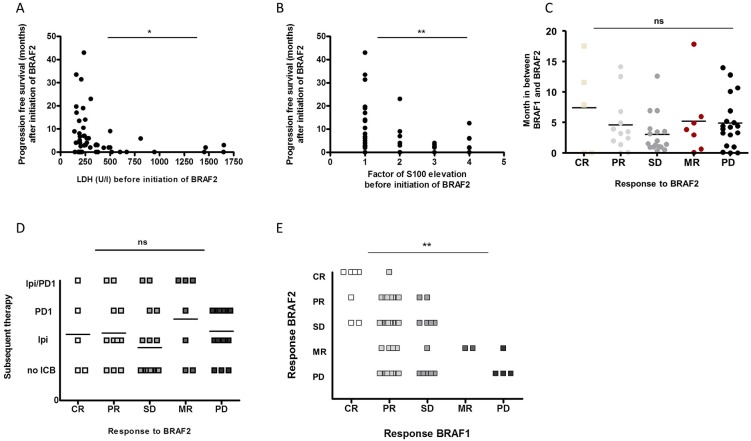
The PFS after BRAF2 correlated with the levels of LDH **(A)** and S100 **(B)** before initiation of BRAF2. Neither the time interval between initial therapy with BRAF1 and re-challenge with BRAF2 **(C)** nor subsequent treatment with ICB **(D)** was correlated with the response to BRAF2. The response to BRAF1 significantly correlated with the response to re-challenge with BRAF2 **(E)**.

We analyzed potentially predictive factors for disease control rate (DCR) under re-challenge with BRAF2. Since S100, LDH and the presence of liver or brain metastases have been associated with overall survival (OS) [18–21], we correlated these factors to the DCR under treatment with BRAF2. However, neither serum levels of S100 nor LDH proved to be prognostic for achieving disease control with BRAF2. There was also no significant correlation between the presence of liver or brain metastases with the rate of disease control under BRAF2. We next analyzed the correlation of these markers to PFS after initiation of BRAF2. Low levels of LDH (p=0.043) and S100 (p=0.012) before initiation of the treatment with BRAF2 were correlated with an increased PFS under BRAF2 (Figure 3A, 3B).

Next we analyzed the correlation of the lengths of the interval between treatment with BRAF1 and BRAF2 to the response to treatment with BRAF2, since the duration of the interval between BRAF1 and BRAF2 has been reported to influence the response to treatment with BRAF2 [12, 14, 16]. However, we could not observe a correlation between the duration of the interval to DCR or PFS and response to treatment with BRAF2 in our cohort (Figure 3C).

It has also been described that subsequent immunotherapy may lead to a higher DCR after re-challenge with BRAF2. In our analysis 67% (40/60) of the patients received subsequent immunotherapy, but no difference in DCR or in PFS to patients without subsequent immunotherapy could be detected (Figure 3D).

Last we correlated the response to BRAF1 with the response to BRAF2 and observed that patients who responded to BRAF1 were significantly more likely to respond to a re-challenge with BRAF2. DCR under BRAF2 was also positively related to the degree of the DCR such as CR, PR or SD under BRAF1 (p=0.007) (Figure 3E); all patients with CR to BRAF1 achieved disease control with BRAF2. Almost all CR (80%, 4/5) to BRAF2 had been CR to BRAF1, and 83% (10/12) of PR to BRAF2 had been PR or CR to BRAF1. Of the patients with PR to BRAF1 only 60% achieved disease control to BRAF2, and only 50% of the patients with SD to BRAF1 achieved disease control with BRAF2 Supplementary Table 1.

### Reduced response by using the same BRAF inhibitor for re-challenge

Most of the analyzed patients (53/60) received two different BRAF inhibitors, while 7 patients were treated with identical BRAF1 and BRAF2. Four of these patients received a combination of BRAF1 with MEK inhibition and after re-challenge with BRAF2 five were treated with an additional MEK inhibitor. All of these seven patients responded well to BRAF1 with 1 CR, 5 PR and 1 SD. But the DCR of these patients after re-challenge with the same BRAF inhibitor as before was low; only 29% (2/7) had SD upon BRAF2 compared to 60% (32/53) DCR in patients treated with distinct BRAF1 and BRAF2 inhibitors (Table 2). The median PFS was also shorter with 5.4 months in patients with identical BRAF1 and BRAF2 compared to 8.9 months in patients with different BRAF1 and BRAF2. However, due to low patient numbers, the difference was not significant.

**Table 2:** Subgroup analysis of patients re-challenged with BRAF inhibitor 2 (BRAF2) identical to BRAF inhibitor 1 (BRAF1) (n=7) in comparison to patients re-challenged with BRAF inhibitor 2 (BRAF2) distinct to BRAF inhibitor 1 (BRAF1) (n=53)

Response to re-challenge		
**BRAF1 identical to BRAF2**	**7**	**12%**
DCR	2	29%
CR	0	0%
PR	0	0%
SD	2	29%
**BRAF1 diverse to BRAF2**	**53**	**88%**
DCR	32	60%
CR	5	9%
PR	12	23%
SD	15	28%

### No difference in PFS and OS after addition of MEK inhibitor to BRAF2

**Figure 4:**
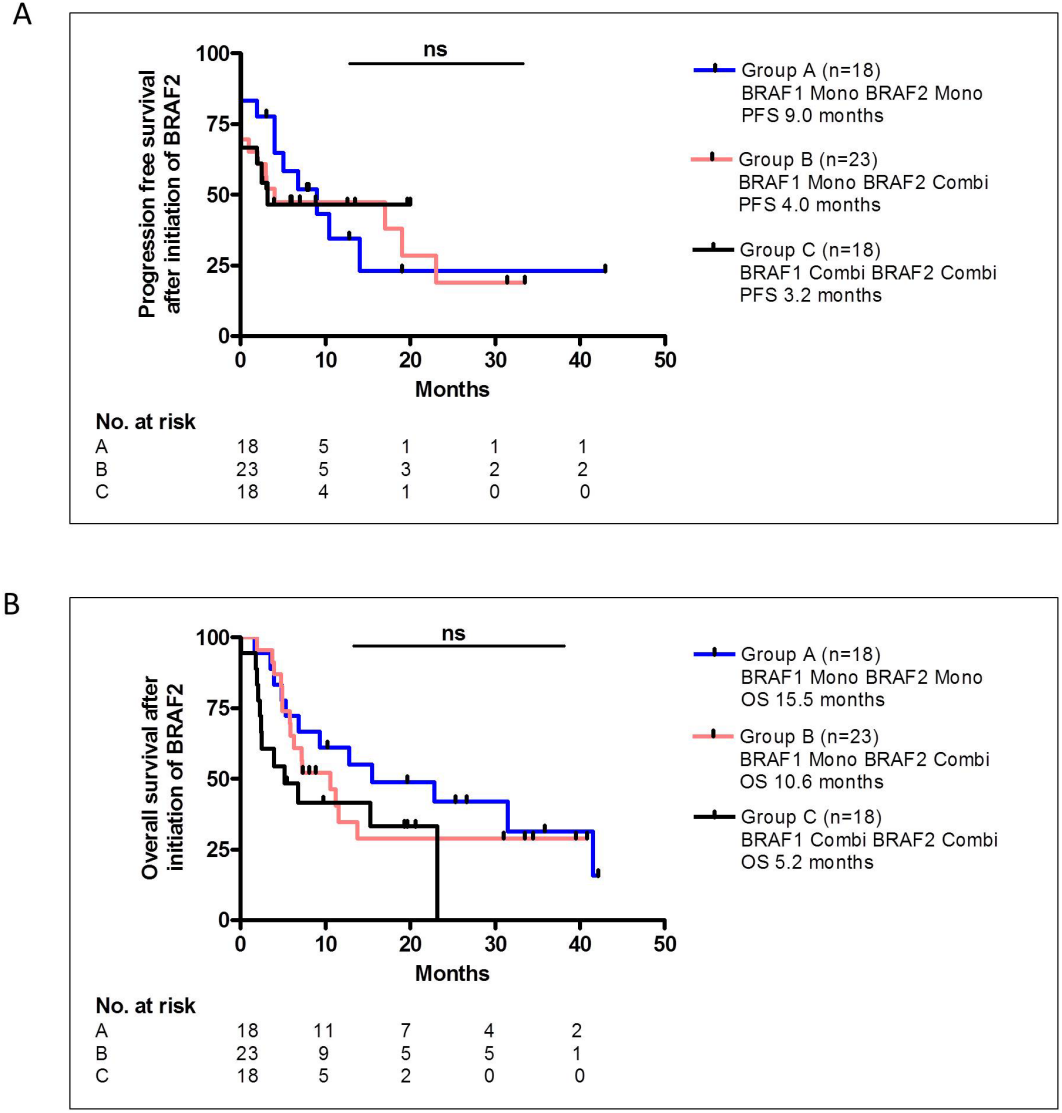
The PFS **(A)** or OS **(B)** after BRAF2 did not differ significantly between patients in Group A, who were treated with BRAF1 and BRAF2 as monotherapy, and patients in Group B, who were treated with BRAF1 as monotherapy and with BRAF2 as a combination therapy with MEK inhibition, and patients in Group C, who were treated with BRAF1 and BRAF2 as a combination therapy with MEK inhibition.

Since only 32% of the patients received an additional MEK inhibitor to treatment with BRAF1, but 68% of patients treated with BRAF2 received additional MEK inhibition, we questioned whether the additional MEK inhibitor contributed to the DCR under treatment with BRAF2. We compared patients who received BRAF1 and BRAF2 as monotherapy (Group A, n=18) with patients who received BRAF1 as monotherapy and BRAF2 as combination therapy with an additional MEK inhibitor (Group B, n=23). Moreover, in the comparison we also included patients who received both BRAF1 and BRAF2 as combination therapy with an additional MEK inhibitor (Group C, n=18). The DCR under BRAF2 did not differ significantly among the groups; in group A the DCR was 67% with 2 CR, 3 PR and 7 SD, in group B the DCR was 52% with 1 CR, 5 PR and 6 SD and in group C the DCR was 56% with 2 CR, 4 PR and 4 SD. PFS of group A was 9.0 months, of group B 4.0 month and of group C 3.2 month and did not differ significantly between the groups (Figure 4A). The OS after initiation of BRAF2 was 15.5 months in group A, 10.6 months in group B and 5.2 months in group C, however, the difference was also not statistically significant (Figure 4B).

## DISCUSSION

Here we present 60 patients with BRAF-V600-mutated melanoma, who had PD upon previous therapy with a BRAF inhibitor (BRAF1) and were re-challenged with another or the same BRAF inhibitor (BRAF2). Despite the previous development of resistance most patients benefited from the re-challenge; the DCR after re-challenge was 57%, with 8% CR, 20% PR and 28% SD.

As mentioned in the introduction so far only a few groups evaluated the efficacy of re-challenge with a BRAF-inhibitor. Here we are discussing our results in context to the findings of Roux et al. [14], Schreuer et al., [15] and Valpione et al. [17].

The duration of the time of the interval between BRAF1 and BRAF2 was hypothesized to be relevant to the response rate to BRAF2, since increasing the time gap between the two courses of BRAF inhibition may restore the driver function of the mutated BRAF oncogene [12, 14]. Valpione et al., indeed observed significantly more responders after a longer treatment interval (8.8 to 6.7 months), however responders were also observed after an interval as short as 0.9 months. However, in our investigations we did not observe any correlation between the duration of the interval between BRAF1 and BRAF2 and the response rate to re-challenge. This finding corresponds to the report of Schreuer et al. and Roux et al., who also could not detect a significant difference in the BRAF inhibitor-free interval of responders and non-responders [15, 17]. In the mouse model of Thakur et al. the regression of BRAF inhibitor-resistant melanoma cells after drug withdrawal was short-lived, i.e., it lasted only for about 10 days [12]. It is therefore possible that a short withdrawal of the BRAF inhibitor after progression will be sufficient to lead to regression of the drug-dependent resistant melanoma cells.

The kind of therapy in the interval between the treatment the with BRAF inhibitors has been observed to be relevant. Immunotherapy subsequent to the first BRAF inhibitor therapy and prior to re-challenge with BRAF inhibitors has been described to increase the response rate by Roux et al.; however, the study population was very small [14]. In our retrospective analysis we could not confirm this result; we did not see any difference in the response rate nor in the duration of response to re-challenge with or without prior immunotherapy. This finding corresponds to the finding of Valpione at al.[17], who could not observe a significant difference with our without IT treatment either. We therefore concluded that the treatment in the interval is not relevant for the response after BRAF2.

The analysis of prospective factors of the DCR or the PFS after initiation of BRAF2 is relevant regarding further treatment decisions and was performed by all groups. We found a significant correlation of the PFS after initiation of BRAF2 with the levels of LDH before treatment start with BRAF2, which is consistent with the data of Valpione et al. and Schreuer et al. [15, 17]. We also found a highly significant correlation between the response of BRAF1 and the response of BRAF2. The higher the tumor reduction after BRAF1 the more likely the patient responded to re-challenge with BRAF2. Valpione et al., observed a similar correlation demonstrating that the duration of response to BRAF1 was significantly correlated to the response rate to BRAF2 (R: 14.8 and NR: 9.7 months).

Furthermore in our study patients who responded to re-challenge with BRAF2 seemed to benefit longer from the treatment, since the DoR of 14.0 months after BRAF2 was significantly higher compared to the DoR of 10.7 months after BRAF1.

It has been shown that BRAFi-resistant cell clones retain sensitivity to MEK inhibition [22]. The response to re-challenge could have therefore been related to the additional benefit of added MEK inhibition. In this retrospective analysis 68% (41/60) of the patients received BRAF1 as monotherapy. However, patients, who received monotherapy and then combination therapy, patients, who were treated solely with monotherapy and patients, who were treated solely with combination therapy, had no significantly different response rate, PFS or OS (Table 2). With an OS of 15.5 months after initiation of BRAF2 patient in group A seemed to be favored; however, this observation may be mainly caused by a shorter observation period of group C, since one third of the patients in group C were still alive at time of analysis. This finding also corresponds to the observation of Schreuer et al. [15]. Valpione et al. though observed a significant difference in the OS after addition of MEK-inhibition with or without LEE1 to BRAF2. However, in these data patients receiving additional LEE1 were also included, which may contribute to the longer survival.

The question whether re-challenge should be performed with the same or a different BRAF inhibitor was not addressed in any study so far. We observed a difference in the response rates of patients re-challenged with the identical BRAF inhibitor (29%) compared to patients re-challenged with a different BRAF inhibitor (60%), but because of the small sample size the difference was not significant. Several mechanisms of BRAF inhibitor resistance have been described, among these alternative splicing isoforms of BRAF [7, 22–26]. So far it has not been shown that mutations in the BRAF domain which interfere with drug binding could contribute to resistance [22, 27] and no data are available on a possible cross-resistance between BRAF inhibitors *in vivo*. Even though the difference in our groups were not significant, it seems worthwhile to exploit the cross-resistance of BRAF inhibitors in prospective studies.

In conclusion this study further supports the observation that re-challenge with BRAF inhibitors despite previous progression upon BRAF inhibition can lead to disease control in over 50% of the cases and therefore should be considered as a reasonable treatment option.

## PATIENTS AND METHODS

### Patients and study design

This study is a retrospective explorative analysis. Inclusion criteria were inoperable stage III or stage IV BRAF V600-mutated melanoma, with progression after previous treatment with a BRAF inhibitor (BRAF1) and re-challenge with the same or another BRAF inhibitor (BRAF2). The records of 60 patients who met the inclusion criteria were investigated. The cases were collected from 13 German skin cancer centers between November 2016 and June 2017. Clinical data and treatment outcomes were extracted from patient records and merged to a central database. Three different combinations of BRAF and MEK inhibitiors were used; dabrafenib 150 mg twice daily and trametinib 2mg once daily, vemurafenib 960 mg twice daily and cobimetinib 60mg once daily for 21 days and 7 day break every month, and encorafenib 450 mg and binimetinib 45 mg twice daily at 100% dosage. When needed the dosage was reduced following the guidelines. This retrospectively collected blinded analysis was conducted in accordance with the principles of the Helsinki Declaration in its revised version from 2013 and was generally approved by the institutional review board of the Medical Faculty of the Munich University Hospital.

### Detection of BRAF mutations

Mutations were tested preferentially in distant or lymph node metastases. If no such samples were available, analyses were performed in primaries. Areas of tumor tissue were identified from 10 μm-thick formalin-fixed, paraffin-embedded sections. To enrich for a content of >75%, the tumors were manually micro-dissected with an ultra-thin cannula. DNA was isolated with extraction buffer (Tris-HCl 0.1 M, EDTA 0.5 mM, Tween 20 0.5% in distilled H_2_O) after proteinase K digestion for 16 h (Thermo Fisher Scientific, Darmstadt, Germany). The samples were amplified with polymerase chain reactions (PCR) covering BRAF exon 15 (Codon 600), NRAS exon 2 (codon 12, 13), and NRAS exon 3 (codon 61). The corresponding primer sequences are displayed in Supplementary Table 1. PCR products were subsequently subjected to pyrosequencing with the PyroMark Q24 System (Qiagen, Hilden, Germany). If the sequencing results were unclear or if mutations were detected in both genes, pyrosequencing was followed by additional Sanger's sequencing to confirm the mutational status which was performed by MWG Operon (Ebersberg, Germany).

### Definitions

*Overall survival (OS):* Time from initiation of treatment until death from any cause.

*Progression-free survival (PFS):* Time from initiation of treatment of all patients until disease progression or death.

*Duration of response (DoR):* Time from documentation of tumor response in responders to disease progression.

*Disease control rate:* All patients with either complete response, partial response or stable disease.

*Complete Response (CR)*: Disappearance of all lesions.

*Partial Response (PR)*: At least a 30% decrease in the sum of the longest diameter (LD) of target lesions, taking as reference the baseline sum LD.

*Stable Disease (SD)*: Neither sufficient shrinkage to qualify for PR nor sufficient increase to qualify for PD, taking as reference the smallest sum LD since the treatment started.

*Mixed response (MR):* shrinkage of some metastases and increase of other metastases.

*Progressive Disease (PD)*: At least a 20% increase in the sum of the LD of target lesions, taking as reference the smallest sum LD recorded since the treatment started or the appearance of one or more new lesions; appearance of new metastases or both.

### Data collection and treatment outcomes

The following clinical data were collected: Eastern Cooperative Oncology Group (ECOG) performance status, BRAF genotype, metastatic sites, number of affected organ systems, serum levels of LDH and S100 and previous systemic therapies. The clinical responses were assessed by the site investigators and indicated as CR, PR, SD and progressive disease (PD+MR) based on the RECIST criteria version 1.1. [28]. Since MR was frequently mentioned in the literature related to re-challenge with BRAF inhibitors, we decided to present this outcome as well, but we did not consider MR as a response based on RECIST nor as controlled disease. Adverse events (AEs) were graded based on the Common Terminology Criteria for Adverse Events (CTCAE) v4.03 published by the National Institutes of Health in 2010. In addition, treatment-related deaths and treatment discontinuation due to severe AEs were reported.

### Statistical analyses

OS was defined as time from initiation of the treatment with BRAF2 until melanoma-related death. Survivors were censored at the time of last documented follow-up. PFS was calculated as time from initiation of the treatment with BRAF1 and BRAF2, respectively, until disease progression determined by imaging. The survival and progression probabilities were calculated with the Kaplan-Meier method assuming proportional hazards. Survival curves were compared with the log-rank test.

Cox proportional hazards regression was applied to correlate the relationship of factors of interest with OS. Cox regression was performed as univariate or multivariate analyses. Hazard ratios (HR) with 95% confidence intervals (CI) were indicated to quantify the impact of a given factor on survival. *P*-values were calculated based on Wald statistics.

Comparisons of variables with treatment groups were performed with the Chi-squared (categorical data) or Kruskal-Wallis (discrete data) test. The association of disease control as dichotomous variable (CR+PR+SD vs. PD+MR) with clinical characteristics or laboratory values was assessed with the Chi-squared test and binary logistic regression for categorical and continuous variables, respectively. Two-tailed *p*-values were calculated and considered significant with values *p*<0.05. All analyses were carried out with SPSS statistics version 23.0 (IBM, Armonk, USA) or GraphPad Prism version 5.01 (GraphPad Software Inc., La Jolla, USA).

## SUPPLEMENTARY MATERIALS TABLE



## Abbreviations

CR: complete response
CI: confidence intervals
DCR: Disease control rate
DoR: duration of response
PDGFR: elevation of platelet-derived growth factor receptor-β
EGFR: epidermal growth factor receptor
HR: Hazard ratios
ICB: immune checkpoint blockade
LDH: Lactate dehydrogenase
PFS: Median progression-free survival
MEK: mitogen-activated protein kinase
MR: mixed responses
NRAS: mutations in N-ras proto-oncogene
OS: overall survival
PR: partial responses
PD-1: Programmed cell death protein 1
BRAF: serine-threonine protein kinase B-RAF
SD: stable diseases
