# Supplementary material for: The efficacy of re-challenge with BRAF inhibitors after previous progression to BRAF inhibitors in melanoma: A retrospective multicenter study
Source: Oncotarget. 2018 Sep 28;9(76):34336–46. doi: 10.18632/oncotarget.26149 (PMC6188134; doi:10.18632/oncotarget.26149)
Supplement: Supplementary file 1 [file oncotarget-09-34336-s001.pdf]

The efficacy of re-challenge with BRAF inhibitors after previous progression to BRAF inhibitors in melanoma: A retrospective multicenter study

SUPPLEMENTARY MATERIALS

Supplementary Table 1: Response to BRAF2 significantly correlated to the degree of response to BRAF1

		CR		PR		SD		MR		PD	
	**Response to BRAF1**	**7**	**12%**	**35**	**58%**	**12**	**20%**	**2**	**3%**	**4**	**7%**
**Response to BRAF2**	CR	4	57%	1	3%	0	0%	0	0	0	0%
	PR	1	14%	9	26%	2	17%	0	0	0	0%
	SD	2	29%	11	31%	4	33%	0	0	0	0%
	MR	0	0%	5	14%	1	8%	2	100%	1	25%
	PD	0	0%	9	26%	5	42%	0	0	3	75%
